# Effects of ADHD Medications on Male Reproductive Function: A Systematic Review of In Vivo Mammalian Evidence

**DOI:** 10.3390/ijms27198492

**Published:** 2026-09-23

**Authors:** Kacper Wojtysiak, Zuzanna Ratka, Agnieszka Chłopaś-Konowałek

**Affiliations:** 1 Scientific Society for Neurotoxicology, Department of Forensic Medicine, Wroclaw Medical University, Mikulicza-Radeckiego 4J, 50-345 Wroclaw, Poland; kacper.wojtysiak@student.umw.edu.pl; 2 Department of Clinical and Experimental Pathology, Wroclaw Medical University, Marcinkowskiego 1, 50-368 Wroclaw, Poland; zuzanna.ratka@student.umw.edu.pl; 3 Department of Forensic Medicine, Division of Molecular Techniques, Wroclaw Medical University, Sklodowskiej-Curie 52, 50-369 Wroclaw, Poland

**Keywords:** ADHD medications, male reproductive health, in vivo mammalian evidence, spermatogenesis, methylphenidate, lisdexamfetamine

## Abstract

Long-term treatment with attention-deficit/hyperactivity disorder (ADHD) medications may begin before reproductive maturity, but their effects on male reproductive health remain uncertain. We systematically evaluated controlled in vivo mammalian evidence on male reproductive effects of medications used for ADHD, including the consistency, timing, reversibility, and certainty of reported associations. PubMed/MEDLINE, Embase, and Scopus were searched from inception to 8 August 2026. Eligible studies were English-language, controlled in vivo mammalian experiments evaluating a prespecified ADHD medication and at least one male reproductive outcome. Human, in-vitro-only, uncontrolled, conference-abstract, and preprint reports were excluded. Risk of bias was independently assessed by two reviewers using the Systematic Review Centre for Laboratory Animal Experimentation (SYRCLE) tool, and certainty was evaluated using an adapted preclinical Grading of Recommendations Assessment, Development and Evaluation (GRADE) framework. Results were grouped by drug and nine prespecified outcome domains and synthesized narratively. Meta-analysis was not performed because of substantial methodological and outcome heterogeneity. Twenty-two studies were included: 19 evaluated methylphenidate (MPH), two clonidine, and one lisdexamfetamine (LDX); 17 were conducted in rats, four in mice, and one in rhesus macaques. During active MPH exposure, adverse changes predominated in spermatogenesis, sperm quantity or quality, testicular or epididymal microstructure, testosterone, and apoptotic or inflammatory markers. Findings after drug-free follow-up were more often neutral, mixed, or partially reversible. Possible persistent sperm DNA fragmentation and adverse paternal embryonic outcomes were reported but were based on few studies with clustering concerns. Clonidine studies indicated altered epididymal contractility and transport, whereas the single high-dose LDX study reported adverse sperm, testicular, endocrine, DNA-fragmentation, and inflammatory findings. Risk-of-bias assessments were dominated by unclear judgments. None of the 18 drug-by-outcome-domain bodies of evidence reached high or moderate certainty; all were rated as very low certainty. Evidence was limited by small samples, heterogeneous species, developmental stages, doses, routes, exposure durations and assays, incomplete numerical reporting, serious indirectness to therapeutic use in men, and sparse replication outside MPH. ADHD medications, particularly MPH during active exposure, show a possible male reproductive toxicity signal in animal models, but the evidence does not permit quantitative risk estimation or class-wide or clinical conclusions. Better-controlled, exposure-validated animal studies and prospective human cohorts are needed.

## 1. Introduction

Attention-deficit/hyperactivity disorder (ADHD) is increasingly diagnosed and treated across age groups, with particularly rapid growth in adult diagnosis and prescribing in several countries [1,2,3,4]. In the United States, approximately 1 in 9 children had ever received an ADHD diagnosis in 2022, corresponding to 7.1 million children [5]. Longitudinal European data likewise show substantial increases in ADHD medication use, with methylphenidate (MPH) remaining one of the most frequently prescribed agents [4,6,7].

Male infertility is also a major and increasing public-health burden. Global burden analyses estimate roughly 55–57 million prevalent cases, with marked growth since 1990 [8,9]. Because ADHD treatment is often initiated during childhood or adolescence and may continue for years, potential effects on male reproductive development and function warrant careful evaluation.

An earlier systematic review synthesized animal studies published through 2016, but numerous subsequent in vivo studies have assessed pubertal development, reproductive hormones, gonadal histology, spermatogenesis, sperm quality and DNA integrity, oxidative and inflammatory mechanisms, and paternal reproductive outcomes. Human evidence remains limited and largely observational. We therefore undertook an independent systematic review of controlled in vivo mammalian studies to characterize the consistency, timing and reversibility, dose- and regimen-dependence, risk of bias, and certainty of male reproductive effects associated with medications used for ADHD [10].

## 2. Methods

### 2.1. Protocol, Registration, and Reporting Framework

This systematic review was designed and reported in accordance with the Preferred Reporting Items for Systematic Reviews and Meta-Analyses 2020 statement (Supplementary Materials S6 and S7) [11]. The eligibility criteria, outcome domains, data items, risk-of-bias procedure, and synthesis plan were established in a working methodological framework before screening commenced. The review was not prospectively registered, and no separate public protocol was published. Prospective registration was not incorporated into the review workflow at study inception; once screening had commenced, we did not retrospectively register the review because retrospective registration would not recreate the principal safeguard of prospective registration. To limit post hoc methodological decisions, the prespecified core methods were maintained throughout the review, standardized extraction forms were used, extracted data were checked by a second reviewer, and no amendments or refinements to the eligibility criteria were required. The study identification, screening, eligibility assessment, and inclusion process was documented using the PRISMA 2020 flow diagram (Figure 1). The database searches identified 315 records (PubMed, *n* = 98; Embase, *n* = 131; Scopus, *n* = 86). After removal of 172 duplicate records, 143 records underwent title and abstract screening. Of these, 119 were excluded, and 24 reports were sought for retrieval and assessed for full-text eligibility. Two reports were excluded at the full-text stage: one because only female animals were exposed while male mating partners remained unexposed, and one because the exposure was performed ex vivo exclusively. Ultimately, 22 studies were included in the systematic review (Figure 1).

### 2.2. Eligibility Criteria

Original controlled in vivo animal studies were eligible when they evaluated principal stimulant and non-stimulant medications used for ADHD, including methylphenidate, dexmethylphenidate, serdexmethylphenidate, amphetamine and its clinically relevant formulations or synonyms (dextroamphetamine, dexamphetamine, d-amphetamine, mixed amphetamine salts, and lisdexamfetamine), atomoxetine (tomoxetine), clonidine, guanfacine or viloxazine, and reported at least one outcome relevant to male reproductive health. No restriction was imposed on animal species, strain, developmental stage, dose, route of administration, exposure frequency, or exposure duration. Single-dose, short-term, and chronic exposure models were therefore eligible. Doses or regimens that differed from clinical practice were not excluded solely for limited clinical comparability; these differences were instead recorded and considered when assessing heterogeneity, indirectness, and applicability.

Eligible comparisons required an ADHD-medication-exposed group and an appropriate untreated, saline, or vehicle control group. Multi-arm studies were retained, and every eligible dose or schedule was extracted separately while the shared control group was preserved as a single group. Its sample size was neither divided nor duplicated, and no combined effect was calculated. Studies evaluating a protective intervention, such as vitamin E, were eligible when they also contained a drug-only arm and a suitable control arm. The primary review comparison was drug alone versus control; findings from drug-plus-protective-intervention arms were retained separately as supplementary evidence and were not treated as estimates of the unmodified drug effect.

Studies including both sexes were eligible when male data were reported separately. Only data for males were extracted, together with embryonic or offspring outcomes attributable to paternal exposure. Embryonic and offspring outcomes were eligible when males had been exposed before mating, and the paternal effect could be distinguished from direct maternal or offspring exposure. Studies in which both parents or the offspring were exposed, and the paternal contribution could not be isolated, were excluded from this outcome category.

A publication containing both in vivo and in vitro or ex vivo experiments was eligible if it included a qualifying in vivo exposure. Measurements performed ex vivo on tissues collected after in vivo drug exposure were retained. Experiments in which isolated tissues or cells were exposed to the drug exclusively in vitro were excluded. The main purpose of the source article did not have to be reproductive toxicology: pharmacokinetic, juvenile-toxicity, developmental, or broader toxicological studies were eligible if they reported at least one qualifying male reproductive outcome.

Human studies, in-vitro-only studies, studies without a relevant male outcome, uncontrolled reports, reviews, editorials, letters without original data, conference abstracts without a full report, and preprints were excluded. Studies of methamphetamine and studies evaluating ADHD medications other than those prespecified above were excluded. Only reports published in English were considered.

### 2.3. Outcome Domains

The review included nine prespecified outcome domains: (1) spermatogenesis and germ-cell indices, including testicular germ-cell numbers and ratios, stage-specific germ-cell differentiation, spermatogenic histological indices, and molecular markers of germ-cell proliferation or maturation; (2) sperm production, count, and quality, including testicular sperm-head counts, homogenization-resistant spermatid counts, daily sperm production, epididymal sperm reserves or concentration, epididymal transit, motility, viability, morphology, and membrane or acrosomal characteristics; (3) testicular and epididymal structure and function, including reproductive-organ development, weight and gross morphology, structural histology or morphometry, and epididymal or vas deferens contractility; (4) reproductive hormones, including testosterone and gonadotropins; (5) sexual maturation, including preputial separation and testicular descent; (6) sperm DNA and chromatin integrity; (7) sexual behavior, mating, and fertility, including sexual or copulatory behavior, mating success, and explicitly reported conception-, pregnancy-, fertility-, or fecundity-level outcomes; (8) embryonic and offspring outcomes after paternal exposure, including preimplantation embryos, implantation, embryonic or fetal loss, resorptions, litter size, fetal or offspring number, sex, weight, morphology, development, and molecular outcomes; and (9) mechanistic reproductive-tissue outcomes related to oxidative stress, antioxidant defenses, apoptosis, or inflammation. Embryonic, blastocyst, fetal, and offspring outcomes were included only when the paternal contribution could be isolated.

Domain assignment followed the operational nature and biological level of the reported endpoint rather than the terminology used by the original investigators. A study contributed to a domain only when at least one corresponding eligible result was reported; outcomes mentioned or defined only in the Methods section of an included study but not subsequently reported in its Results section or supplementary materials were not counted. A study could contribute to more than one domain but was counted only once within each drug-by-domain body of evidence.

Within each domain, individual outcomes were retained separately rather than collapsed into a reviewer-created composite. Statistical significance was not an eligibility or prioritization criterion; significant, non-significant, neutral, and discordant findings were extracted according to the same rules.

### 2.4. Information Sources and Search Strategy

PubMed/MEDLINE, Embase, and Scopus were searched from database inception to the final search on 8 August 2026. Database-specific strategies combined controlled vocabulary, where available, with free-text terms for the prespecified drugs and terms describing male reproduction, fertility, testes, epididymides, spermatogenesis, sperm, sexual maturation, and paternal reproductive outcomes. The complete line-by-line strategies can be found in Supplementary Material S1, and search dates and numbers retrieved from each database are reported in Section 2.4 and in Figure 1, respectively.

Supplementary files associated with eligible articles were assessed whenever available. No study authors were contacted, and unpublished numerical results were not requested.

### 2.5. Selection Process

Search results were imported into Rayyan and deduplicated [12]. Titles and abstracts were screened manually against the prespecified criteria, followed by full-text assessment of potentially eligible records. Title and abstract screening was performed independently by two reviewers. Any disagreements were resolved through discussion until consensus was reached. Eligibility decisions were based on the complete report and its supplementary material where available. Reasons for exclusion at the full-text stage were recorded and are presented in the PRISMA flow diagram and in Section 3.1. No automation tool was used to make inclusion or exclusion decisions.

When several publications described the same experiment, animal cohort, or overlapping material, they were linked and treated as a single study. Complementary information from all related reports was used, but the same animals and outcomes were not counted more than once. Any discrepancies between linked reports were recorded. The analytical unit for synthesis was therefore the experiment or study rather than the publication.

### 2.6. Data Collection Process and Data Items

Data were extracted manually using prespecified, standardized forms for study characteristics and outcome-level results. Main articles and all available supplementary materials were examined. Extracted entries were checked against their source reports before synthesis. No automated extraction system was used. Data were extracted by one reviewer and subsequently checked and confirmed by a second reviewer. Any discrepancies or uncertainties were resolved through discussion.

Study-level items comprised authorship and year, country or setting when reported, study aim, animal species and strain, sex, age or developmental stage, body weight, group allocation and sample size, drug and formulation, dose, route, frequency and duration of exposure, timing of outcome assessment, washout or follow-up, comparator, additional intervention, funding source, ethics approval and declared conflicts of interest. Missing information was recorded as not reported rather than inferred.

Outcome-level extraction captured the outcome definition and measurement method, tissue or biological material, analysis time point, intervention and comparator groups, group sample sizes, reported numerical values, units, measures of variability, reported effect measures, direction of effect and statistical significance. Data were preserved in the form reported by the authors. Units were not converted, missing effect sizes or confidence intervals were not calculated, and no reviewer-generated pooled or standardized estimate was produced. Values were not digitized from figures. If a graph or narrative statement supported only the direction and/or statistical significance of an effect, the finding was retained qualitatively and explicitly marked as lacking complete numerical data.

For embryonic or offspring observations clustered within the same sire or litter, data were extracted exactly as analyzed by the investigators. The unit of analysis and any reported accommodation of sire- or litter-level clustering were recorded. No independent statistical correction was performed. Failure to account for non-independence was considered a potential source of pseudoreplication and was incorporated into risk-of-bias and certainty judgments.

### 2.7. Risk-of-Bias Assessment

Risk of bias was assessed independently by two reviewers with the SYRCLE risk-of-bias tool for animal intervention studies, with any disagreements resolved through discussion and consensus [13]. Ten domains were evaluated: sequence generation, baseline characteristics, allocation concealment, random housing, blinding of caregivers and investigators, random outcome assessment, blinding of outcome assessors, incomplete outcome data, selective outcome reporting, and other potential sources of bias. Each domain was judged as low, high, or unclear risk of bias, with a study-specific rationale based only on the main report and supplementary materials.

Selective reporting was assessed by comparing outcomes and analyses described in the Methods sections of the included studies with those reported in their Results sections and supplementary materials. Absence of a publicly available protocol was treated as a source of uncertainty but did not automatically produce a high-risk judgment. Funding or industry involvement was recorded and considered in interpretation, but commercial funding alone was not treated as evidence of high risk across all domains. Studies were not excluded from the synthesis solely because they had high or unclear risk of bias; instead, their limitations were reflected in the interpretation and certainty assessment.

### 2.8. Effect Measures and Preparation of Results

Effect measures were retained as reported by the investigators, including means with standard deviations or standard errors, medians and ranges, proportions, ratios, coefficients, and *p*-values. Direction of effect was assigned only from reported drug-versus-control comparisons and was interpreted according to the biological meaning of the endpoint. Missing effect sizes or confidence intervals were not calculated, and dose–response patterns were described only when supported by reported between-group findings.

### 2.9. Synthesis Methods and Assessment of Heterogeneity

A structured narrative synthesis was undertaken because substantial clinical and methodological heterogeneity was expected across animal species and strains, ages at exposure, dose levels, administration routes, treatment schedules, follow-up periods, reproductive domains, assays, and units of measurement. Studies were grouped first by drug and then by outcome domain. Within each group, the direction, magnitude as reported, statistical significance, timing, and consistency of findings were compared. Individual measurements within a domain remained visible in the outcome tables, whereas domain-level conclusions were formulated qualitatively without constructing a composite score.

No meta-analysis was performed; therefore, no pooled effect estimate, I^2^ statistic, formal heterogeneity test, funnel plot, Egger test, quantitative subgroup analysis, or formal sensitivity analysis was calculated. Potential sources of heterogeneity were explored narratively by comparing drug, species and strain, developmental stage, dose, route, exposure duration, follow-up, model characteristics, and outcome measurement method. The influence of studies with high or unclear risk of bias, atypical regimens, small samples, or limited clinical relevance was considered qualitatively.

### 2.10. Reporting-Bias Assessment

Risk of missing evidence and publication bias was assessed qualitatively within each body of evidence. Judgments considered the number and size of available studies, the predominance of positive findings, incomplete numerical reporting, selective outcome reporting, and the absence of unpublished data. Funnel plots and statistical tests for small-study effects were not used because the evidence bodies were small and methodologically heterogeneous.

### 2.11. Certainty of Evidence

Certainty was evaluated using an adapted preclinical GRADE framework informed by Hooijmans et al. (2018) [14]. A body of evidence was defined as a specific drug-by-outcome-domain combination. Experimental animal evidence was initially considered at high certainty within the adapted framework and could be downgraded for risk of bias, inconsistency, indirectness, imprecision, or publication bias; final ratings were high, moderate, low, or very low. Because the framework was developed principally for preclinical intervention evidence rather than reproductive toxicology, ratings were interpreted as transparent review-level judgments about the credibility and reproducibility of the direction of association in animal models, not as direct clinical certainty in men.

Risk of bias was downgraded when a substantial proportion of the relevant evidence came from studies with serious or multiple unclear methodological limitations. Inconsistency was judged from agreement in direction and biological interpretation across studies while considering species, strain, age, dose, regimen, and measurement method. Indirectness addressed the translational relevance of the model, developmental stage, dose, route, exposure duration, and outcome to therapeutic use in men. Imprecision was judged from animal numbers, number of studies, variability, completeness of numerical data, and stability of the conclusion. Publication bias was judged qualitatively as described above.

Upgrading was considered only for a convincing large effect, a consistent dose-response gradient, or a situation in which plausible residual confounding would be expected to attenuate rather than create the observed effect. Missing reporting contributed only to the domain directly affected to avoid double counting. Certainty findings are presented in Supplementary Material S4.

### 2.12. Deviations, Missing Data, and Transparency

Missing values were not imputed, graph-only values were not digitized, and no reviewer-generated statistical estimates were created. Prespecified handling rules for mixed-sex reports, paternal exposure, protective-intervention arms, linked reports, ex vivo measurements after in vivo exposure, shared controls, and atypical dosing regimens were applied consistently to avoid double counting. No formal review protocol was registered or published. The prespecified eligibility criteria and review methods described above were applied without subsequent amendment or refinement.

## 3. Results

### 3.1. Study Selection

A total of 22 studies met the eligibility criteria and were included in the systematic review. The complete study identification and selection process is presented in the PRISMA 2020 flow diagram in Section 2 (Figure 1).

Two reports were excluded after full-text assessment. Asir et al. was excluded because the investigated drug was administered only to female animals, while the male mating partners remained unexposed [15]. The study by da Silva Júnior et al. was excluded because clonidine exposure was performed ex vivo exclusively in isolated rat testicular tissue rather than in vivo [16]. The final evidence set comprised 22 full-text articles and one supplementary file linked to Mattison et al. (2011); the article and supplement were treated as a single study [17].

### 3.2. Characteristics of the Included Studies

The 22 included studies were published between 2002 and 2025. Nineteen evaluated methylphenidate (MPH), including one study of racemic d,l-MPH, two evaluated clonidine, and one evaluated lisdexamfetamine (LDX). Seventeen studies used rats, four used mice, and one used rhesus macaques. The models covered juvenile, prepubertal, peripubertal, adolescent, young-adult, and adult exposure. Group sizes were generally small and varied by outcome; several experiments used 6–18 animals per group, the paternal-fertility component of Fazelipour et al. (2012) [18] used three males per group, and the largest juvenile-toxicity study enrolled approximately 38–41 animals of each sex per group, with smaller pathology and fertility subsets. One report did not provide a readily extractable group size for the reproductive endpoints.

Exposure regimens were highly diverse. MPH was administered predominantly by oral gavage, although intraperitoneal dosing and dietary administration were also used. Exposure ranged from a single dose or 14-day adolescent course to approximately 40 months in rhesus macaques. Nominal MPH doses ranged from 2 mg/kg/day in several rodent experiments to 100 mg/kg/day in the juvenile rat study; dietary concentrations in Manjanatha et al. [19] corresponded to estimated doses of approximately 8–142 mg/kg/day depending on study week, and the macaque regimen was escalated to 12.5 mg/kg twice daily. The single LDX study used 30 mg/kg/day by oral gavage for four weeks. The clonidine studies evaluated either an acute intravenous dose–response experiment or an escalating intraperitoneal regimen over 7–9 days. Follow-up ranged from immediate end-of-exposure assessment to approximately two months after treatment cessation, while the primate study included repeated measurements across development.

The most frequently assessed domain was testicular and epididymal structure or function (20 studies), followed by spermatogenesis (12 studies), sperm count and quality (12 studies), and reproductive hormones (12 studies). Eight studies reported mechanistic tissue outcomes related to oxidative stress, apoptosis, or inflammation. Sexual maturation and paternal embryonic or offspring outcomes were each examined in four studies, sexual behavior, mating, or fertility in three, and sperm DNA or chromatin integrity in two. Protective-intervention arms involving vitamin E, ascorbic acid, or curcumin and nicotine-containing combination arms were retained in the extraction but were not used to estimate the primary effect of the ADHD medication alone. Detailed study characteristics are provided in the accompanying tables (Table 1A,B), and detailed outcome-level data that were extracted are provided in Supplementary Material S2. Ethics approval, funding, and conflict-of-interest reporting in the included studies were assessed and can be found in Supplementary Material S8.

### 3.3. Risk of Bias

Across the 22 studies and 10 SYRCLE domains, 55 of 220 judgments (25.0%) were low risk, 157 (71.4%) were unclear risk, and eight (3.6%) were high risk. No study was judged to be at low risk across all domains. Seven studies had at least one high-risk judgment. Cansu et al. (2011) [20] and Mattison et al. (2011) [17] had the largest number of low-risk judgments, with four of ten each; no other study had more than three.

Sequence generation was judged low risk in one study, high risk in one, and unclear in the remaining 20. Baseline comparability was low risk in 16 studies and unclear in six. Random outcome assessment was unclear in all 22 studies because none adequately reported random selection or ordering of animals or animal-level samples during outcome assessment.

Reporting was particularly limited for safeguards against selection and performance bias. Allocation concealment, random housing, and blinding of caregivers or investigators were unclear in all 22 studies. Blinding of outcome assessors was explicitly supported only in Cansu et al. (2011) [20], in which two blinded observers assessed TUNEL and immunohistochemical outcomes. Selective reporting was unclear in every study because no prospective protocol or registration allowed the complete prespecified outcome set to be verified.

Incomplete outcome data were judged low risk in 21 studies and high risk in Beckman et al. (2008) [21], where deaths and variable subset sizes made the effect of attrition on reproductive analyses uncertain. Six studies were judged high risk for other bias because internal discrepancies, probable tabulation or unit errors, or an unclear unit of analysis could materially affect interpretation: Akhavan Rezayat et al. (2020), Fazelipour et al. (2012), Fazelipour et al. (2014), Gomes et al. (2022), Kianifard et al. (2013), and Raoofi et al. (2021) [18,22,23,24,25,26]. These concerns included conflict between narrative and tabulated directions, values or units that appeared implausible, inconsistent group labels, unreconciled sample sizes, and contradictory reporting of sperm motility. Such findings were retained as reported and were not corrected or imputed by the reviewers. Study-level SYRCLE judgments, supporting rationales, and summary counts are provided in Supplementary Material S5.

### 3.4. Effects of Methylphenidate

#### 3.4.1. Spermatogenesis and Reproductive-Tissue Structure

Eleven MPH studies contributed to the spermatogenesis domain. During ongoing exposure, the predominant pattern was adverse. Abdollahifar et al. (2020) [27] reported fewer spermatogonia, primary spermatocytes, spermatids, and Leydig cells after 5 mg/kg/day for 30 days. Cansu et al. (2011) [20] found lower round-spermatid-to-Sertoli-cell ratios, particularly at 10 mg/kg/day, and Basaki et al. (2021) [28] reported reductions in selected tubular differentiation and repopulation indices and in germ-cell or Sertoli-cell counts. Akhavan Rezayat et al. (2020), Iqbal et al. (2025), Raoofi et al. (2021), and Rodrigues et al. (2025) [23,26,29,30] likewise described impaired germ-cell maturation, reduced proliferative or spermatogenic markers, or lower Sertoli- and Leydig-cell indices. Fazelipour et al. (2014) [24] reported a mixed result, with increased spermatogonial counts but lower epithelial height and ambiguity between the narrative and table annotations.

Time since exposure modified this pattern. Kianifard et al. (2013) [22] observed reduced tubular diameter, epithelial height, tubular differentiation index, and spermiogenesis index after continuous or intermittent exposure, whereas the group exposed for two weeks and then followed for nine weeks showed improvement. Khoubbieh et al. (2023) [31] reported tubular atrophy, vacuolization, reduced epithelial thickness, and loss of the spermatogenic cell line during developmental exposure, with histological appearance approaching that of controls after a 30-day recovery period. At approximately 40 days after juvenile exposure, Montagnini et al. (2014) [32] found mostly neutral germ-cell results with isolated, bidirectional differences in stage VII cell populations. Thus, recovery studies generally suggested at least partial reversibility, although complete normalization was not demonstrated for every structural endpoint.

Seventeen MPH studies assessed testicular or epididymal structure or function. Recurrent findings included lower testicular volume or weight, reduced seminiferous-tubule or epithelial dimensions, interstitial expansion, Leydig-cell depletion, vacuolization, and germ-cell disorganization. These effects were often dose- or time-dependent rather than uniform across all measurements. Akhavan Rezayat et al. (2020), Cansu et al. (2011), Iqbal et al. (2019), Iqbal et al. (2025), Raoofi et al. (2021), and Rodrigues et al. (2025) [20,23,26,29,30,33] reported significant structural abnormalities during or shortly after exposure. Manjanatha et al. (2008) [19] found dose-related reductions in seminal-vesicle weight and changes in testicular weight only at high dietary concentrations. In contrast, Beckman et al. (2008) [21] found no MPH-related reproductive-organ pathology at the end of juvenile treatment or after recovery, and Kianifard et al. (2013) [22] found no significant change in absolute or relative testicular weight despite histological and spermatogenic effects.

The longitudinal primate study provided evidence of developmental delay rather than persistent adult injury. High-dose MPH reduced testicular volume at selected months and delayed testicular descent, but these differences resolved by later assessments. Khoubbieh et al. (2023) [31] similarly found no significant change in reproductive-organ weights at PND60, PND90, or after recovery despite clear transient histological changes. Montagnini et al. (2014) [32] reported increased interstitial-tissue volume after washout, while most organ-weight and tubular measures were unchanged. Collectively, gross organ weight was less consistently affected than histology or morphometry.

#### 3.4.2. Sperm Quantity, Quality, and Chromatin Integrity

Ten MPH studies evaluated sperm count or quality. Reduced sperm count, motility, viability, or normal morphology was reported by Abdollahifar et al. (2020), Akhavan Rezayat et al. (2020), Basaki et al. (2021), Kianifard et al. (2013), Raoofi et al. (2021), and Rodrigues et al. (2025) [22,23,26,27,28,29], although the affected parameter and magnitude differed substantially. Rodrigues et al. found lower spermatid production and epididymal reserves together with prolonged transit time. The direction of the motility finding in Raoofi et al. was internally contradictory and was therefore considered uncertain, while the reductions in count, viability, and normal morphology were retained as adverse.

Several studies showed neutral or transient rather than persistent effects. Adriani et al. (2006) [34] reported a transient increase in total sperm-head count after 14 days, but the difference disappeared after normalization to testicular weight and after a two-month recovery. Mattison et al. (2011) [17] found no sustained difference in sperm concentration; high-dose animals showed altered head morphology at one assessment, without a persistent adult difference. Montagnini et al. (2014) [32] found no effect on spermatid counts, daily sperm production, epididymal reserves, motility, or viability after washout, although abnormal tails increased at 2.5 mg/kg but not at 5 mg/kg. Gomes et al. (2022) [25] found no change in acrosome integrity after a 39-day washout.

Gomes et al. (2022) [25] was the only MPH study to assess sperm DNA and chromatin directly. Sperm chromatin structure assay measures of DNA fragmentation increased after the 39-day washout, whereas chromomycin A3 staining, high DNA stainability, and acrosome integrity did not differ significantly. Because the numerical values were available only in graphs, the direction and reported significance were retained without digitization.

#### 3.4.3. Reproductive Hormones and Sexual Maturation

Eleven MPH studies reported reproductive endocrine outcomes. Testosterone was lower during or shortly after exposure in Akhavan Rezayat et al. (2020), Fazelipour et al. (2012), Fazelipour et al. (2014), Raoofi et al. (2021), and Rodrigues et al. (2025). Adriani et al. (2006) [18,23,24,26,29,34] found reduced intratesticular testosterone after normalization to testicular weight following 14 days of exposure, with no residual effect after recovery. In rhesus macaques, Mattison et al. (2011) [17] reported lower testosterone across selected developmental months in both dose groups, but not at the final adult assessments. Basaki et al. (2021), Khoubbieh et al. (2023) [28,31], and Montagnini et al. (2014) [32] found no significant testosterone difference for the relevant MPH-versus-control comparisons, while group labeling and significance reporting in Kianifard et al. (2013) [22] precluded a confident directional conclusion. FSH and LH were generally unchanged or inconsistent; isolated increases in LH or inhibin B did not form a reproducible pattern.

Four MPH studies examined sexual maturation including three rodent studies and one longitudinal rhesus macaque study. Preputial separation was unchanged in Beckman et al. (2008) [21] and Montagnini et al. (2014) [32], whereas Khoubbieh et al. (2023) [31] observed a small but statistically significant advance in the mean day of preputial separation. Mattison et al. (2011) [17] reported delayed testicular descent in the high-dose macaque group, but all testes had descended by month 30. The available evidence therefore did not indicate a consistent direction for pubertal timing and primarily reflected model-specific, transient findings. It is worth acknowledging that, given this sparse and heterogeneous evidence base regarding sexual maturation, the MPH findings did not support a consistent developmental signal, and the rhesus macaque findings are considered separately in the developmental interpretation below.

#### 3.4.4. Sexual Behavior, Fertility, and Outcomes After Paternal Exposure

Three MPH studies directly assessed sexual behavior, mating, or fertility. Beckman et al. (2008) [21] found 100% mating in all groups and no significant dose-related difference in fertility or fecundity after at least five weeks of recovery. Montagnini et al. (2014) [32] found no effect on the reported sexual-interest or copulatory-behavior measures after juvenile exposure and washout. In contrast, Fazelipour et al. (2012) [18] reported a dose-related reduction in fertility, from 95.4% in controls to 65.0% and 18.5% at 2 and 10 mg/kg, respectively. That result was based on only three exposed males per group, and the reported fertility measure was defined as embryos per corpora lutea rather than a conventional pair-level conception rate.

Four studies contributed paternal embryonic or offspring outcomes. Fazelipour et al. (2012) [18] also entered this domain because its reported “fertility rate” was calculated as embryos per corpora lutea; the endpoint decreased with MPH, but only three exposed males contributed to each mating group and the result overlaps conceptually with the direct-fertility finding above. Beckman et al. (2008) [21] found no significant changes in corpora lutea, implantations, preimplantation loss, or early resorptions. Gomes et al. (2022) [25], after a 39-day paternal washout, reported more asymmetric or fragmented early embryos and fewer high-quality symmetric embryos. Implantation and pre- or postimplantation loss were unchanged, but litter size and the numbers of male and female fetuses were reduced, while fetal weight was unaffected. Gomes et al. (2025) [35] subsequently found markedly poorer blastocyst quality after paternal MPH exposure, with no difference in the proportion reaching the blastocyst or morula stage and with changes in H3K9me3 and H4K20me3 but not H3K4me3. These embryo-level analyses involved observations clustered within sires and females; incomplete reporting of clustering adjustment limited confidence in the nominal precision of the results.

#### 3.4.5. Oxidative Stress, Apoptosis, and Inflammation

Six MPH studies evaluated mechanistic tissue outcomes. Abdollahifar et al. (2020) [27] and Cansu et al. (2011) [20] reported increased TUNEL-positive cells, while Basaki et al. (2021) [28] found increased tissue malondialdehyde and p53 immunoreactivity at the higher dose. Raoofi et al. (2021) [23] reported coordinated increases in inflammatory mediators and pro-apoptotic proteins together with reductions in anti-apoptotic proteins. These findings were broadly compatible with apoptosis and inflammatory injury, but many magnitudes were graph-only, and the assays differed substantially between studies.

The redox findings were less uniform. After a 39-day washout, Gomes et al. (2022) [25] found no increase in testicular or epididymal malondialdehyde and observed higher glutathione, suggesting an adaptive antioxidant response rather than persistent lipid peroxidation. Rodrigues et al. (2025) [29] reported lower superoxide dismutase and malondialdehyde with no change in catalase, glutathione S-transferase, or nitric oxide; this pattern did not support a simple interpretation of increased oxidative damage. Accordingly, the mechanistic evidence was more consistent for apoptosis and inflammation than for a uniform oxidative-stress signature.

### 3.5. Effects of Clonidine

Two rat studies evaluated clonidine and focused principally on epididymal transport physiology. In the acute intravenous experiment by Chaturapanich et al. (2002) [36], clonidine increased baseline and total cauda epididymal pressure and increased contraction frequency without significantly changing contraction amplitude; yohimbine abolished the response, supporting mediation through α_2_-adrenoceptors. This experiment demonstrated an acute functional effect on epididymal contractility rather than chronic reproductive toxicity.

After an escalating intraperitoneal regimen over 7–9 days, da Silva Júnior et al. (2014) [37] reported lower testis, epididymis, and prostate weights, reduced caput/corpus and cauda sperm reserves, accelerated epididymal transit, and increased spontaneous cauda epididymal contraction frequency and amplitude. Daily sperm production and testicular spermatid counts were not significantly altered. Sperm lipid peroxidation and glutathione peroxidase activity increased, whereas superoxide dismutase activity decreased. Thus, the clonidine evidence converged on altered epididymal contractility and transport, but it derived from only two parenteral regimens with distinct acute and short-term designs.

### 3.6. Effects of Lisdexamfetamine

The LDX evidence consisted of one study in adult male rats exposed to 30 mg/kg/day by gavage for four weeks (Roustaee et al., 2024) [38]. Total and progressive motility decreased, non-progressive motility decreased, and the immotile fraction increased; sperm count and the proportion with normal morphology showed non-significant decreases. DNA fragmentation increased, whereas aniline-blue staining of chromatin immaturity was unchanged. Testosterone, spermatogonial, primary-spermatocyte, spermatid, and Leydig-cell measures, seminiferous-tubule length, and the Johnsen score were reduced, while Sertoli-cell counts were unchanged. The study also reported adverse testicular geometric changes and increased caspase-3 and TNF-α. Although the direction was internally coherent across several domains, the absence of replication and the high nominal dose precluded a stable conclusion about LDX or about clinically relevant exposure.

### 3.7. Heterogeneity and Suitability for Quantitative Synthesis

Marked heterogeneity in drug, species and strain, developmental stage, dose, route, exposure duration, washout, outcome definition, assay, unit, and analytical level precluded meaningful quantitative pooling. The evidence ranged from acute clonidine physiology to developmental MPH models and a 40-month macaque study, while several outcomes were available only as graphs or lacked directly comparable effect estimates and confidence intervals. End-of-exposure and post-washout findings were kept separate because several studies showed recovery. Accordingly, no meta-analysis or statistical heterogeneity estimate was performed. Study-level characteristics underlying the qualitative assessment of heterogeneity are summarized in Supplementary Material S3.

### 3.8. Certainty of the Evidence

Certainty ratings addressed the reproducibility and credibility of associations in animal models, not direct clinical certainty in men. Eighteen drug × outcome bodies of evidence were assessed: nine for MPH/d,l-MPH, three for clonidine, and six for LDX. None achieved high or moderate certainty. All 18 bodies of evidence were rated as very low certainty.

Risk of bias was serious across evidence bodies because key safeguards such as allocation concealment, random housing, and blinding were usually unreported. Inconsistency reflected discordant, transient or reversible, and endpoint-dependent findings and was not assessable for single-study bodies. Indirectness was generally serious and was very serious for paternal embryonic or offspring outcomes. Imprecision was serious or very serious because of small samples, few contributing studies, graph-only data, absent confidence intervals, heterogeneous measures, and clustering concerns. Publication bias could not be excluded, and no upgrading criterion was applied. Within these constraints, MPH showed the most reproducible adverse signal during active exposure, whereas clonidine and LDX remained too sparsely studied for stable drug-specific conclusions. Domain-specific judgments and rationales are provided in Supplementary Material S4.

## 4. Discussion

### 4.1. Principal Findings and Interpretation

This systematic review synthesized 22 in vivo studies of male reproductive outcomes after exposure to medications used for ADHD. The evidence base was dominated by MPH (19 studies), with only two clonidine studies and one LDX study. Across the nine prespecified outcome domains, the clearest qualitative signal was a pattern of adverse changes during active MPH exposure involving spermatogenesis, sperm quantity or quality, testicular and epididymal microstructure, testosterone, and markers of apoptosis or inflammation. Magnitude, dose–response, and persistence varied substantially, and none of the drug × outcome evidence bodies reached moderate or high certainty. These findings therefore indicate a possible reproductive hazard in animal models rather than a quantified effect or evidence of impaired fertility in men.

Microscopic and functional abnormalities were more consistent than changes in gross reproductive-organ weight. Seminiferous epithelial thinning, altered tubular dimensions, germ-cell disorganization, vacuolization, and reduced Sertoli- or Leydig-cell indices recurred in several studies despite neutral organ weights. Gross organ weight therefore appears to be an insensitive surrogate in this literature and should not be interpreted as evidence that reproductive structure or function was preserved.

Endocrine findings likewise suggested a predominantly testicular rather than consistently central neuroendocrine pattern. Testosterone was reduced in several MPH experiments during or shortly after exposure and was sometimes accompanied by lower Leydig-cell numbers or interstitial abnormalities, whereas FSH and LH were usually unchanged or inconsistent. This pattern is compatible with impaired steroidogenic capacity or local testicular injury but does not establish a single mechanism. Several testosterone differences were no longer present after recovery, including in the longitudinal rhesus macaque study, arguing against a uniform persistent disruption of the hypothalamic–pituitary–gonadal axis.

Mechanistic findings partly supported the biological plausibility of the structural and sperm effects. Increased TUNEL staining, p53-related responses, caspase activation, pro-apoptotic signaling, and inflammatory mediators recurred across several MPH studies, and the single LDX study showed a broadly concordant phenotype. Redox findings were less uniform, with both pro-oxidative and adaptive or mixed antioxidant patterns. The evidence therefore supports apoptosis and inflammation more consistently than a simple model of sustained oxidative damage. Protective-intervention arms were not used to estimate the primary drug effect and do not establish a clinically effective protective strategy.

### 4.2. Mechanistic Framework

The available evidence supports a multi-pathway mechanistic framework rather than a single established molecular cascade. MPH increases catecholaminergic signaling primarily through inhibition of the dopamine transporter (DAT) and norepinephrine transporter (NET) [39]. Catecholamines can also modulate hypothalamic–pituitary–gonadal signaling and local testicular pathways, and dopaminergic and adrenergic receptors have been identified in several testicular cell populations [40]. However, none of the included reproductive studies directly demonstrated that MPH-induced gonadal injury was initiated by local DAT or NET inhibition or directly linked a measured change in catecholaminergic signaling to a downstream reproductive lesion. These pathways should therefore be regarded as biologically plausible upstream mechanisms rather than experimentally established mediators within the present evidence base.

Downstream reproductive-tissue findings provide stronger mechanistic evidence because several components were directly measured in the included studies. MPH exposure was associated with increased TUNEL staining, p53-related responses, pro-apoptotic signaling, and inflammatory mediators in several experiments [20,23,28], while the single LDX study reported increased caspase-3 and TNF-α together with impaired germ-cell and Leydig-cell measures, lower testosterone, and adverse sperm outcomes [38]. These observations are compatible with a testicular-injury phenotype involving apoptotic and inflammatory signaling, although they do not establish a single causal sequence. Redox findings were less uniform: lipid-peroxidation or antioxidant abnormalities were reported in some experiments, whereas others showed adaptive or discordant responses [25,28,29]. Oxidative stress should therefore be considered a context-dependent component rather than a universally demonstrated intermediate. Mitochondrial function itself was not directly assessed in the included studies and should not be presented as an established mediator.

Clonidine provides a more directly localized mechanistic pathway. α_2_-Adrenoceptors are G-protein-coupled receptors that couple predominantly to inhibitory G_i_/G_o_ proteins, classically reducing adenylyl-cyclase-dependent cyclic adenosine monophosphate signaling and also modulating Ca^2+^ and K^+^ channels [41]. In the included epididymal experiments, clonidine-induced contractile effects were sensitive to α_2_-adrenoceptor antagonism, while repeated or in vivo exposure was associated with altered spontaneous cauda epididymal contractility, accelerated sperm transit, reduced epididymal sperm reserves, and altered sperm oxidative markers [36,37]. However, neither included study directly measured intracellular cyclic adenosine monophosphate signaling or ion-channel activity in the epididymis; the intracellular step linking α_2_-adrenoceptor activation or desensitization to altered contractility therefore remains unresolved. Altered epididymal smooth-muscle physiology nevertheless provides a plausible functional bridge between clonidine exposure and changes in sperm transport and maturation.

A distinct germline pathway may involve sperm DNA and chromatin integrity. Following adolescent MPH exposure and a 39-day washout, Gomes et al. (2022) [25] reported increased sperm DNA fragmentation together with adverse paternal reproductive outcomes, while Gomes et al. (2025) [35] subsequently reported poorer blastocyst quality accompanied by alterations in H3K9me3 and H4K20me3, but not H3K4me3. These observations raise the possibility that molecular sperm abnormalities may persist despite normalization of some conventional reproductive measures. However, the evidence derives from very few experiments, and the observed associations do not establish a causal epigenetic mechanism. A graphical outline of the mechanistic framework discussed in this section can be found below (Figure 2).

### 4.3. Developmental Stage, Timing, Reversibility, and Paternal Effects

Developmental stage is particularly important when interpreting reversibility. In sexually mature animals, treatment-related changes occur after the major developmental establishment of the Sertoli-cell population and seminiferous architecture. By contrast, depending on the species and exact exposure window, prepubertal or peripubertal exposure may coincide with late Sertoli-cell proliferation and/or functional maturation and with ongoing maturation of the seminiferous epithelium. The final Sertoli-cell population is an important determinant of adult testicular size and sperm-supporting capacity [42,43]. Species differences in the timing of these processes are substantial: rodents undergo rapid postnatal reproductive development and lack the prolonged juvenile testicular quiescence characteristic of humans and other primates, limiting direct age-for-age extrapolation of developmental exposure windows [43,44]. The single rhesus macaque study is therefore particularly informative for developmental timing, although it remains insufficient for a primate-specific conclusion; importantly, the reported reductions in testicular volume and delay in testicular descent were transient rather than clearly persistent [17]. Accordingly, the present evidence concerning sexual maturation should be regarded as sparse, species-specific, and insufficient to establish permanent developmental gonadotoxicity.

Assessment timing was one of the clearest effect modifiers. During ongoing or recently completed MPH exposure, adverse histological, spermatogenic, sperm, and testosterone findings predominated. After a drug-free interval, results were more often neutral, mixed, or partially reversible. Improvement was reported after short developmental exposure followed by recovery, juvenile exposure followed into adulthood, and repeated longitudinal assessment in macaques. These findings favor transient developmental delay or reversible functional suppression over inevitable permanent gonadotoxicity for several conventional endpoints. Nevertheless, this interpretation remains provisional because washout duration was inconsistent, did not always span a complete spermatogenic cycle, and was confounded with dose, age at exposure, and study design. Studies with recovery periods were not randomized comparisons of discontinuation versus continued exposure, and the observed contrast cannot be attributed to washout alone.

The principal exception to the generally more reassuring postrecovery pattern concerned sperm DNA integrity and paternal embryonic outcomes. After a 39-day washout, Gomes et al. reported increased sperm DNA fragmentation, poorer early-embryo morphology, reduced litter size, and subsequently poorer blastocyst quality with changes in selected histone marks. These observations raise the possibility that conventional semen parameters may normalize while molecular sperm abnormalities or paternal effects persist. They are biologically important because DNA or chromatin integrity and embryo competence are closer to reproductive success than organ weight alone. At the same time, this was one of the least certain parts of the evidence base: only two studies assessed sperm DNA or chromatin directly, paternal outcomes were reported in four studies, and embryo- or blastocyst-level observations were clustered within a small number of sires and females. Incomplete adjustment for this non-independence may have overstated statistical precision. These findings should therefore be treated as a high-priority signal for replication, not as established evidence of transgenerational toxicity.

Direct fertility evidence was similarly insufficient to bridge the gap between adverse surrogate outcomes and reduced reproductive success. A small mouse experiment reported a pronounced dose-related reduction in an embryos-per-corpora-lutea measure, but only three exposed males contributed to each mating group, and the reported endpoint was not a conventional pair-level conception rate. Conversely, a larger juvenile rat study reported no dose-related impairment of mating, fertility, fecundity, implantation, or early resorption after recovery, and another postwashout study found no change in sexual or copulatory behavior. Thus, recurrent abnormalities in tissue, hormones, or semen cannot be assumed to translate into infertility, and the available direct reproductive outcomes remain very uncertain.

### 4.4. Drug-Specific Interpretation

The predominance of MPH studies means that the overall narrative largely describes MPH rather than ADHD pharmacotherapy as a class. Even for MPH, nominal doses could not be interpreted as equivalent exposures across gavage, diet, and intraperitoneal administration, and several regimens were substantially higher or more intensive than typical therapeutic exposure. Apparent dose–response relationships within individual studies increased plausibility in some instances, but non-monotonic or isolated findings were also common. Differences between species, maturation rates, first-pass metabolism, treatment schedules, and systemic exposure prevent a direct conversion of mg/kg doses to clinical risk.

The clonidine evidence suggested a mechanistically distinct pathway. Acute clonidine increased cauda epididymal pressure and contraction frequency through an α_2_-adrenoceptor-sensitive mechanism, while short-term escalating exposure increased spontaneous epididymal contractions, accelerated transit, and reduced epididymal sperm reserves without reducing daily sperm production. This convergence supports altered epididymal transport as a plausible clonidine-related effect rather than primary suppression of spermatogenesis. However, it rests on only two rat studies using intravenous or intraperitoneal exposure and cannot establish the consequences of chronic oral treatment.

The LDX evidence was limited to one high-dose rat study. Its internally coherent pattern-reduced motility, increased DNA fragmentation, lower testosterone and germ-cell measures, adverse testicular geometry, and increased caspase-3 and TNF-α justify further investigation. It cannot, however, support a drug-specific risk estimate, a class effect for amphetamine-based stimulants, or an inference about therapeutic exposure. The near absence of replicated evidence for LDX and the lack of eligible evidence for several other ADHD medications are major evidence gaps rather than evidence of safety.

For contextual interpretation only, approximate human-equivalent doses (HEDs) were calculated by body-surface-area scaling using the species conversion factors provided in U.S. Food and Drug Administration guidance (HED = animal dose × animal K_m/human K_m; K_m values: mouse, 3; rat, 6; monkey, 12; adult human, 37) [45]. Under this approach, rat MPH regimens of 2–5 mg/kg/day correspond to HEDs of approximately 0.32–0.81 mg/kg/day, equivalent to approximately 19–49 mg/day in a 60 kg adult. At the opposite extreme, 100 mg/kg/day of MPH in rats corresponds to approximately 16.2 mg/kg/day (approximately 973 mg/day for a 60 kg adult), while the highest estimated dietary mouse exposure of approximately 142 mg/kg/day corresponds to approximately 11.5 mg/kg/day (approximately 691 mg/day). The high-dose rhesus macaque regimen of 12.5 mg/kg twice daily corresponds, on dosing days, to approximately 8.1 mg/kg/day (approximately 486 mg/day), and the 30 mg/kg/day rat LDX regimen corresponds to approximately 4.86 mg/kg/day (approximately 292 mg/day). These estimates demonstrate the wide translational range of the included regimens and the clearly toxicological character of several upper-dose experiments. However, body-surface-area scaling is an approximate dose-scaling method rather than a pharmacokinetic exposure equivalence and does not account for route or formulation, oral bioavailability, first-pass metabolism, active metabolites, dosing schedule, peak concentration, total systemic exposure, developmental stage, or species-specific pharmacodynamics. Moreover, the FDA framework was developed for estimating starting doses in adult humans from animal toxicology data and should not be interpreted as a pediatric dose conversion or as direct evidence of clinical reproductive risk [45].

### 4.5. Comparison with Previous Animal and Human Evidence

The present synthesis both agrees with and qualifies the earlier systematic review by Danborg et al. (2017) [10]. That review included 17 animal studies across both sexes and concluded broadly that ADHD drugs impaired reproduction, while noting poor reporting, substantial heterogeneity, greater effects with earlier exposure, and improvement after drug-free follow-up. Our male-specific, outcome-level synthesis confirms the recurring themes of methodological weakness, heterogeneity, and possible reversibility, but it does not support an equally broad class-level conclusion. The current evidence is dominated by MPH; direct fertility and sexual-maturation outcomes are inconsistent, and clonidine and LDX are represented by only two and one study, respectively. The addition of sperm DNA, paternal embryo-quality, inflammatory, and apoptotic outcomes also shifts attention from conventional reproductive indices toward molecular and intergenerational endpoints, although these newer signals currently have very low certainty.

Human evidence remains sparse and conflicting. In a retrospective analysis of 9769 semen samples from men undergoing infertility investigation, Shalev et al. (2021) [46] found no adverse association between MPH purchasing and conventional semen parameters; current and past users instead had higher values for several count and motility measures, although only 83 current users and 293 past users were identified and prescription purchases did not confirm ingestion. Pham et al. (2022) [47] associated recent stimulant prescriptions with lower semen volume and lower total motile sperm count, without differences in concentration, motility percentage, or morphology. Aliakbari et al. (2022) [48] reported worse count, motility, and abnormality measures among current or previous long-term MPH users. More recently, Siva et al. (2026) [49] found an adjusted 8.4% lower semen volume in 388 stimulant-exposed men with ADHD versus 776 matched unexposed men, with no significant differences in other semen parameters. Taken together, these observational studies neither reproduce a uniformly adverse pattern nor establish a causal effect on human fertility.

Human endocrine data are likewise mixed. Wang et al. (2019) [50] found no significant increase in diagnosed testicular dysfunction with short- or long-term MPH treatment in a nationwide cohort of boys with ADHD, and Wang et al. (2021) [51] found no material disruption of expected gonadal-hormone or Tanner-stage trends during 12 months of usual-dose MPH. By contrast, Ostdiek-Wille et al. (2024) [52] associated long-term stimulant exposure with a higher relative rate of a subsequent testicular-hypofunction diagnosis, although the absolute difference was small and residual confounding remained possible. Differences in age, exposure definition, outcome ascertainment, and confounding control prevent firm clinical conclusions.

The translational gap is therefore substantial. The included animal studies used healthy mammals rather than animals with an ADHD-like phenotype, and many employed early developmental windows, forced dosing routes, or nominal doses that do not closely mimic therapeutic use. Conversely, most animal exposures were brief relative to years of treatment in humans, and clinical studies rarely measured adherence, cumulative dose, sperm DNA integrity, repeated hormonal trajectories, or actual conception and live-birth outcomes. Animal studies may consequently overestimate risk because of dose and route while simultaneously underestimating risk from long cumulative exposure. The direction and magnitude of translational bias cannot be assumed.

### 4.6. Strengths and Limitations

A major strength of this review is its focused evaluation of male reproductive outcomes across 22 in vivo studies and nine prespecified domains. Study characteristics, outcome-level findings, recovery timing, funding, conflicts of interest, and methodological concerns were extracted systematically from the full texts and the available supplement. Drug-only comparisons were distinguished from antioxidant, vitamin, curcumin, or nicotine combination arms. End-of-exposure and postwashout findings were not treated as equivalent, which prevented transient developmental changes from being conflated with persistent injury. The review also avoided false numerical precision: graph-only values were not digitized, unclear or internally contradictory source data were retained as reported, and no unreported values were imputed. Finally, SYRCLE assessment and an adapted outcome-level GRADE framework made the limitations of the animal evidence explicit rather than allowing a predominance of statistically significant findings to be mistaken for high certainty.

Several limitations constrain the review-level conclusions. First, clinical and methodological heterogeneity precluded meta-analysis. Species, strain, developmental stage, dose, route, treatment duration, recovery time, assay, and analytical unit varied widely; even nominally similar endpoints were reported in incompatible units. The narrative synthesis therefore describes patterns in direction and timing rather than pooled effect sizes and remains vulnerable to selective reporting and the unequal number of outcomes contributed by individual studies. Formal statistical heterogeneity, small-study effects, and publication bias could not be assessed.

Second, risk-of-bias judgments were dominated by insufficient reporting. Across the 220 SYRCLE domain assessments, 71.4% were unclear, 25.0% were low risk, and 3.6% were high risk. Allocation concealment, random housing, and caregiver or investigator blinding were unclear in every study, and assessor blinding was explicitly supported in only one. Unclear ratings do not prove that safeguards were absent, but they prevent confidence that observed effects were protected from selection, performance, and detection bias. Seven studies had at least one high-risk judgment, including attrition concerns, internal discrepancies, probable unit or tabulation errors, and uncertain units of analysis. Selective reporting remained unclear for all studies because prospective protocols were unavailable.

Third, the evidence base was imbalanced and often underpowered. Nineteen of twenty-two studies evaluated MPH, while clonidine and LDX had minimal replication and other commonly used ADHD medications were not represented. Group sizes were generally small, and direct mating or paternal-outcome components sometimes relied on very few sires. Analyses conducted at the level of sperm, tubules, embryos, or blastocysts may have produced artificially narrow uncertainty if clustering within animals, females, or litters was not modeled. Confidence intervals were uncommon, graph-only reporting was frequent, and several publications contained contradictory labels, values, or directions.

Fourth, external validity to treated men is limited. Animal developmental stages do not map precisely to pediatric, adolescent, or adult clinical use; pharmacokinetic equivalence was seldom demonstrated; parenteral, dietary, and gavage regimens were not directly comparable; and the studies did not model the physiology or comorbidity profile of ADHD. Conventional semen and histological outcomes were much more common than direct fertility, sperm DNA, offspring health, or multi-generational outcomes. Recovery was assessed in only a subset of studies and often at a single time point. Consequently, the review cannot identify a human no-effect threshold, compare agents, determine whether effects are fully reversible, or estimate the probability of infertility.

Fifth, the review was restricted to English-language full-text original studies and excluded conference abstracts and preprints. This improved verifiability but may have introduced language and availability bias. Grey literature and unpublished regulatory datasets were not incorporated, authors were not contacted, and missing data were neither imputed nor estimated from figures. No formal protocol was publicly registered or published; consequently, external verification that all analytical decisions were fixed in advance is not possible. Publication bias could not be excluded for any outcome domain.

### 4.7. Implications for Research and Practice

Future animal studies should prioritize clinically interpretable exposure rather than another isolated high-dose toxicity experiment. Dose selection should be supported by plasma exposure or pharmacokinetic bridging; oral regimens should reproduce clinically plausible schedules; and developmental exposure should be separated prospectively from adult exposure. Experiments should include adequate sample-size justification, concealed randomization, randomized housing and outcome assessment, blinded analysis, preregistration, and complete reporting aligned with ARRIVE 2.0 [53], including transparent flow of animals and samples. A core outcome set should combine testicular histology, standardized sperm concentration, motility and morphology, serum and intratesticular hormones, sperm DNA integrity, and direct mating outcomes. Recovery should be measured at multiple prespecified time points extending through at least one complete spermatogenic cycle.

Paternal-effect studies require particular methodological improvement. The sire or mating unit, not each embryo or blastocyst, should define biological replication unless hierarchical models explicitly account for embryos nested within females and sires. Replication should assess fertilization, implantation, fetal development, live birth, postnatal health, and whether molecular alterations in sperm or embryos persist across generations. The emerging histone-mark and DNA-fragmentation signals should be reproduced by independent laboratories with orthogonal assays and prespecified primary outcomes. Comparative studies of MPH, LDX and non-stimulant medications are also needed; absence of published evidence for an agent should not be interpreted as absence of reproductive effects.

In humans, prospective longitudinal cohorts should obtain semen and hormonal measurements before treatment, during well-characterized exposure, and after discontinuation or dose modification, while recording adherence, cumulative dose, formulation, smoking, body mass index, comorbidities, concomitant medicines, abstinence interval, fever, and other major fertility determinants. Studies should distinguish drug-specific effects from ADHD-related or behavioral confounding and should extend beyond semen analysis to time to pregnancy, assisted-reproduction outcomes, pregnancy loss, and offspring outcomes after paternal exposure. Until such evidence is available, the present animal data support scientific vigilance and targeted investigation, but not routine discontinuation of effective ADHD medication or universal fertility monitoring. Any clinical decision in a patient with infertility, pubertal concern, or symptoms of hypogonadism should be individualized and based on direct clinical evaluation rather than extrapolation from animal toxicity alone.

## 5. Conclusions

The available in vivo evidence indicates a possible male reproductive toxicity signal for ADHD medications, driven primarily by MPH studies and expressed most consistently during active exposure as adverse spermatogenic, sperm, testicular, testosterone, apoptotic, and inflammatory changes. Many conventional outcomes improved or normalized after treatment cessation, whereas possible persistent sperm DNA and paternal embryo-quality effects remain an important but very-low-certainty signal. Evidence for clonidine and LDX is too sparse for stable drug-specific conclusions, and the data do not justify a class-wide inference. High heterogeneity, predominantly unclear risk of bias, small samples, limited direct fertility outcomes, and serious indirectness to humans prevent quantitative or clinical risk estimation. Well-controlled, exposure-validated animal studies and prospective human reproductive cohorts are required before these findings can inform routine prescribing or fertility counseling.

## Figures and Tables

**Figure 1:**
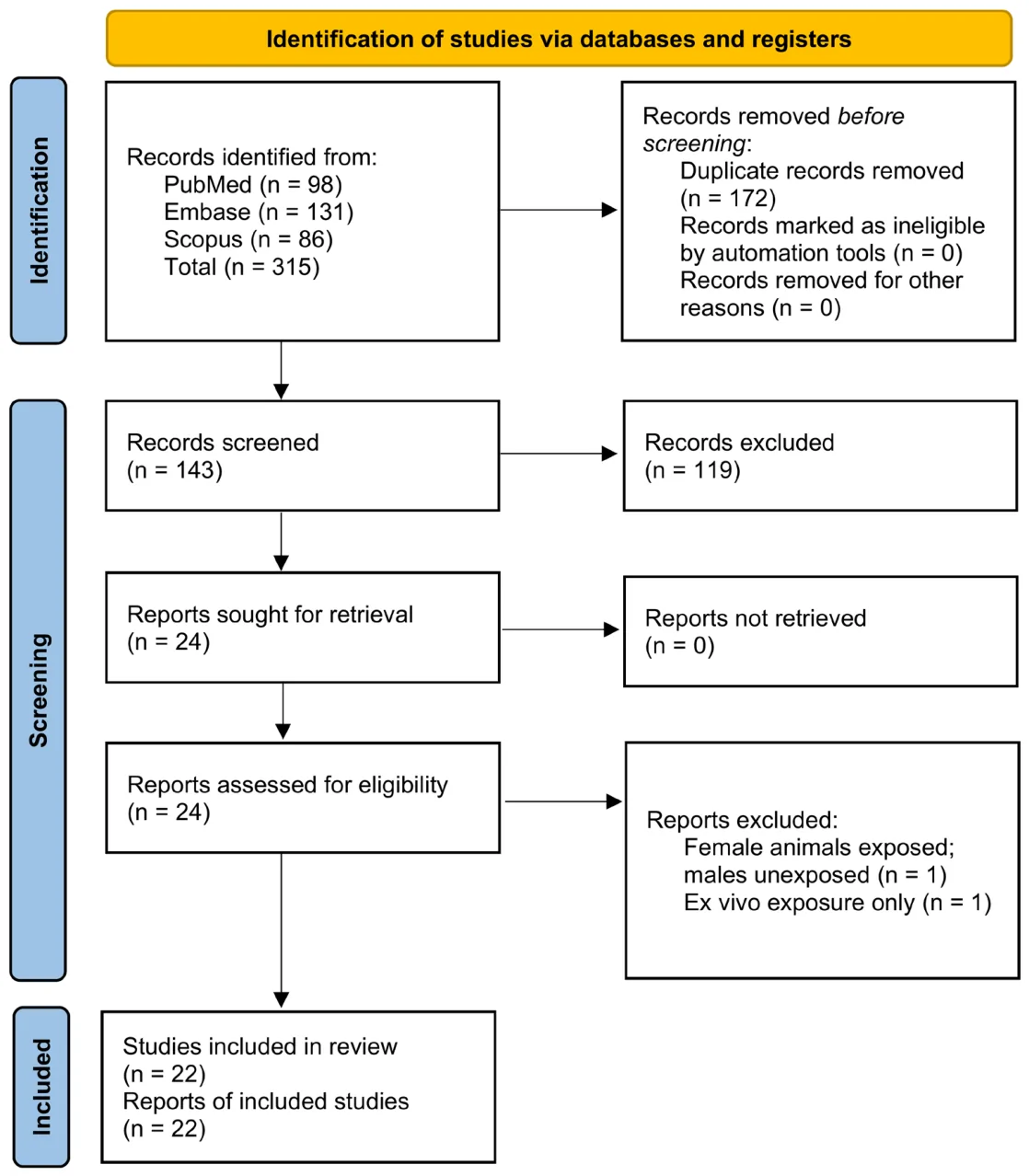
PRISMA 2020 flow diagram of study identification, screening, eligibility assessment, and inclusion.

**Figure 2:**
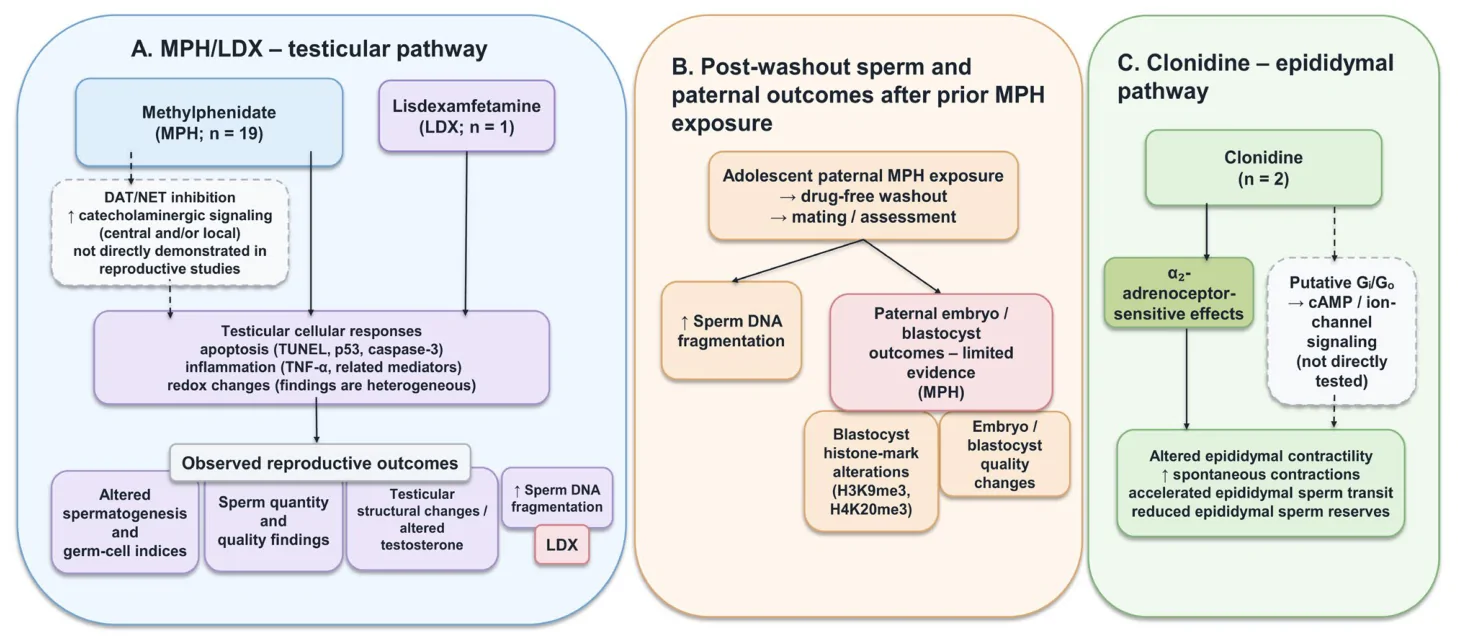
Conceptual mechanistic framework linking ADHD-medication exposure to male reproductive findings identified in the included in vivo mammalian studies. (**A**) MPH- and LDX-associated testicular findings, including apoptotic and inflammatory responses, heterogeneous redox alterations, changes in spermatogenesis and germ-cell indices, sperm quantity or quality, testicular structure, testosterone, and, for LDX, increased sperm DNA fragmentation. For MPH, dopamine and norepinephrine transporter inhibition and consequent catecholaminergic modulation are presented as upstream pharmacological mechanisms but were not directly linked to reproductive injury in the included experiments. (**B**) Postwashout sperm and paternal findings following prior adolescent MPH exposure, including increased sperm DNA fragmentation, altered embryo or blastocyst quality, and changes in blastocyst H3K9me3 and H4K20me3. (**C**) Clonidine-associated epididymal findings, including α_2_-adrenoceptor-sensitive alterations in contractility, accelerated epididymal sperm transit, and reduced epididymal sperm reserves; G_i_/G_o_–cAMP and ion-channel signaling are shown only as plausible intracellular mechanisms because they were not directly assessed in the included studies. Solid connectors indicate associations or linked findings directly supported by the included in vivo evidence, whereas dashed connectors indicate mechanistic links not directly demonstrated in the included reproductive studies. Connections summarize the organization of the evidence and do not by themselves establish causal mediation between intermediate and downstream findings. Abbreviations: DAT, dopamine transporter; LDX, lisdexamfetamine; MPH, methylphenidate; NET, norepinephrine transporter; TNF-α, tumor necrosis factor alpha.

**Table 1:** (**A**) Animal models and study design of the included studies; (**B**) Exposure regimens, comparators, follow-up, and outcomes assessed.

(**A**)			
**Study**	**Drug**	**Animal Model**	**Sample Size**
Mattison et al., 2011 [17]	MPH	Male Indian-origin rhesus macaques, approximately 2 years old	30; 10/group
Fazelipour et al., 2012 [18]	MPH	Male BALB/c mice, 8 weeks old (25–30 g)	27; 9/group
Manjanatha et al., 2008 [19]	MPH	Male B6C3F1 mice, approximately 6 weeks old	60; 10/group
Cansu et al., 2011 [20]	MPH	Prepubertal male Wistar rats, PND21–24	42 randomized; 35 analyzed (12/11/12)
Beckman et al., 2008 [21]	Racemic d,l-MPH	Wistar Hannover rats exposed from PND7; male outcomes extracted	Main cohort ~38–41/sex/group; pathology cohort 12/sex/group
Kianifard et al., 2013 [22]	MPH	Adult male Wistar rats (187 ± 8.96 g)	32; 8/group (one death in continuous-exposure group)
Raoofi et al., 2021 [23]	MPH	Adult male rats, 10 weeks old (strain inconsistently reported as Wistar/Sprague Dawley; 200 ± 20 g)	24; 6/group
Fazelipour et al., 2014 [24]	MPH	Adult male mice, 90 days old (strain not reported)	36; 12/group
Gomes et al., 2022 [25]	MPH	Male Wistar rats exposed from 6 weeks of age; unexposed females used for mating	24 males (12/group) and 32 females
Akhavan Rezayat et al., 2020 [26]	MPH	Male Wistar rats, 8 weeks old (~250 g)	54; 18/group
Abdollahifar et al., 2020 [27]	MPH	Adult male Sprague Dawley rats (200–220 g)	30; 15/group
Basaki et al., 2021 [28]	MPH	Adult male Wistar rats, 70–80 days old	90; nine groups, 10/group
Rodrigues et al., 2025 [29]	MPH	Adult male BALB/c mice, 80 days old (35 ± 10 g)	24; 8/group
Iqbal et al., 2025 [30]	MPH	Male albino Sprague Dawley rats, 10–12 weeks old (180–200 g)	30; 10/group
Khoubbieh et al., 2023 [31]	MPH	Male Sprague Dawley rats exposed from PND21	35; 7/group
Montagnini et al., 2014 [32]	MPH	Male Wistar rats exposed from PND21	78; 26/group (subgroups 11–15)
Iqbal et al., 2019 [33]	MPH	Male albino rats, 8 weeks old (180–200 g; strain not reported)	24; 8/group
Adriani et al., 2006 [34]	MPH	Adolescent male rats (strain not reported)	NR
Gomes et al., 2025 [35]	MPH	Male Wistar rats exposed during adolescence; unexposed females used for mating	21 males (10 control, 11 MPH) and 48 females
Chaturapanich et al., 2002 [36]	Clonidine	Sexually mature male Wistar rats (250–350 g)	63 total; clonidine experiments *n* = 15 and *n* = 5
da Silva Júnior et al., 2014 [37]	Clonidine	Adult male Wistar rats, 90–120 days old (300–400 g)	34 in-vivo-treated rats (17/group); sperm analyses 6/group
Roustaee et al., 2024 [38]	LDX	Adult male albino rats (200–220 g; strain not reported)	24; 12/group
(**B**)			
**Study**	**Exposure Regimen**	**Comparator and Follow-Up**	**Outcome Domains**
Mattison et al., 2011 [17]	Orally twice daily, 5 days/week for 40 months; escalated to 2.5 or 12.5 mg/kg/dose	Control; testes/hormones months 14–40 and semen months 27–38	2, 3, 4, 5
Fazelipour et al., 2012 [18]	2 or 10 mg/kg once daily for 40 days; administration route ambiguously reported	Saline control; assessment after 40 days; mating with unexposed females for 10 days	3, 4, 7, 8
Manjanatha et al., 2008 [19]	250–4000 ppm in diet for 28 days (~8–142 mg/kg/day)	0 ppm control; assessed after 28 days	3
Cansu et al., 2011 [20]	5 or 10 mg/kg/day by oral gavage in two divided doses for 3 months	Water control; assessed after 90 days	1, 3, 9
Beckman et al., 2008 [21]	5, 50, or 100 mg/kg/day by gavage from PND7 to ~PND70	0 mg/kg control; end of dosing, ≥5-week recovery, and fertility from ≥16 weeks	3, 5, 7, 8
Kianifard et al., 2013 [22]	10 mg/kg by oral gavage; 2-week, 11-week continuous, or 11-week intermittent regimens	Control; all groups assessed 11 weeks after study initiation	1, 2, 3, 4
Raoofi et al., 2021 [23]	20 mg/kg/day by gavage for 21 days	Control; MPH vs. control used; assessed after 21 days	1, 2, 3, 4, 9
Fazelipour et al., 2014 [24]	2 or 10 mg/kg by oral gavage once daily for 40 days	Saline control; assessed after 40 days	1, 3, 4
Gomes et al., 2022 [25]	5 mg/kg by gavage once daily on PND38–68	Distilled-water control; 39-day washout; assessment/mating around PND107–130	2, 6, 8, 9
Akhavan Rezayat et al., 2020 [26]	2 or 10 mg/kg by gavage once daily for 60 days	Control group; assessed on day 61	1, 2, 3, 4
Abdollahifar et al., 2020 [27]	5 mg/kg orally once daily for 30 days	Saline control; assessed after 30 days	1, 2, 3, 9
Basaki et al., 2021 [28]	5 or 10 mg/kg i.p. once daily for 8 weeks	Control; MPH-only groups used; assessed after 8 weeks	1, 2, 3, 4, 9
Rodrigues et al., 2025 [29]	20 or 40 mg/kg/day by gavage in two divided doses for 42 days	Water control; assessed after 42 days	1, 2, 3, 4, 9
Iqbal et al., 2025 [30]	2 mg/kg/day orally for 50 days	Saline control; MPH vs. saline used; euthanasia on day 60	1, 3
Khoubbieh et al., 2023 [31]	5 mg/kg/day by oral gavage on PND21–60 or PND21–90	Age-matched controls; PND60/90 assessment and 30-day recovery	1, 3, 4, 5
Montagnini et al., 2014 [32]	2.5 or 5 mg/kg by gavage on PND21–60	Water control; assessed at PND100 after ~40-day washout	1, 2, 3, 4, 5, 7
Iqbal et al., 2019 [33]	10 mg/kg/day by gavage for 40 days	Saline control; MPH vs. saline used; assessed after 40 days	3
Adriani et al., 2006 [34]	2 mg/kg i.p.; single dose or once daily on PND30–44	Saline control; 2 h post-dose or after 2-month recovery	2, 3, 4
Gomes et al., 2025 [35]	5 mg/kg by gavage once daily on PND38–68	Distilled-water control; 39-day washout; mating PND107–130; blastocysts at 4.5 dpc	8
Chaturapanich et al., 2002 [36]	1–100 µg/kg i.v. acutely; selected findings at 10 µg/kg	Within-animal baseline and antagonist controls; acute postdose assessment	3
da Silva Júnior et al., 2014 [37]	Escalating i.p. regimen: 1 mg/kg/day (days 1–2), 3 mg/kg/day (days 3–4), 5 mg/kg/day (days 5–9)	Vehicle control; euthanasia on days 7–9; only findings from the in vivo component extracted	2, 3, 9
Roustaee et al., 2024 [38]	30 mg/kg/day by oral gavage for 4 weeks	Oral saline control; assessed after 4 weeks	1, 2, 3, 4, 6, 9

Abbreviations: LDX, lisdexamfetamine; MPH, methylphenidate; NR, not reported; PND, postnatal day; dpc, days post coitum; i.p., intraperitoneal; i.v., intravenous. Outcome domains: 1, spermatogenesis; 2, sperm count and quality; 3, testicular and epididymal structure or function; 4, reproductive hormones; 5, sexual maturation; 6, sperm DNA and chromatin integrity; 7, sexual behavior, mating, and fertility; 8, embryonic or offspring outcomes after paternal exposure; 9, oxidative-stress, apoptosis, or inflammation mechanisms.

## Data Availability

No new data were created or analyzed in this study. Data sharing is not applicable to this article.

## Supplementary Materials

The following supporting information can be downloaded at: https://www.mdpi.com/article/10.3390/ijms27198492/s1.

## Abbreviations

The following abbreviations are used in this manuscript:

ADHD	attention-deficit/hyperactivity disorder
ARRIVE	Animal Research: Reporting of In Vivo Experiments
d,l-MPH	racemic d,l-methylphenidate
DAT	dopamine transporter
DNA	deoxyribonucleic acid
dpc	days post coitum
FSH	follicle-stimulating hormone
GRADE	Grading of Recommendations Assessment, Development and Evaluation
HED	human-equivalent dose
H3K4me3	trimethylation of lysine 4 on histone H3
H3K9me3	trimethylation of lysine 9 on histone H3
H4K20me3	trimethylation of lysine 20 on histone H4
I2	I-squared statistic
i.p.	intraperitoneal
i.v.	intravenous
LDX	lisdexamfetamine
LH	luteinizing hormone
MEDLINE	Medical Literature Analysis and Retrieval System Online
MPH	methylphenidate
NET	norepinephrine transporter
NR	not reported
p53	tumor protein p53
PND	postnatal day
ppm	parts per million
PRISMA	Preferred Reporting Items for Systematic Reviews and Meta-Analyses
SYRCLE	Systematic Review Centre for Laboratory animal Experimentation
TNF-α	tumor necrosis factor alpha
TUNEL	terminal deoxynucleotidyl transferase-mediated deoxyuridine triphosphate nick-end labeling
