# Supplementary material for: Effects of ADHD Medications on Male Reproductive Function: A Systematic Review of In Vivo Mammalian Evidence
Source: Int J Mol Sci. 2026 Sep 23;27(19):8492. doi: 10.3390/ijms27198492 (PMC13665539; doi:10.3390/ijms27198492)
Supplement: Supplementary file 1 [file ijms-27-08492-s001.zip › Supplementary Material S1.pdf]

Scopus

(TITLE-ABS
(methylphenidate OR dexmethylphenidate OR serdexmethylphenidate OR amphetamin
e OR dextroamphetamine OR dexamphetamine OR "d-amphetamine" OR "mixed
amphetamine
salts" OR lisdexamfetamine OR atomoxetine OR tomoxetine OR clonidine OR guanfacin
e OR viloxazine) AND TITLE-ABS
(sperm* OR spermat* OR testis OR testes OR testicular OR epididym* OR seminiferous
OR Leydig OR Sertoli OR "male fertility" OR "male infertility" OR "male reproductive
toxicity" OR "male reproductive function" OR "male reproductive system" OR (
(male OR males OR paternal OR sire OR sires) AND
(fertil* OR fecund* OR mating OR impregn* OR "sexual maturation" OR "preputial
separation"))) AND TITLE-ABS (rat OR rats
OR mouse OR mice OR murine OR rabbit* OR hamster* OR "guinea pig" OR "guinea
pigs" OR monkey* OR primate* OR canine OR dog OR dogs OR pig OR pigs OR swine))

Pubmed

("Methylphenidate"[Mesh] OR methylphenidate[tiab] OR dexmethylphenidate[tiab] OR serdexmethylphenidate[tiab] OR "Amphetamine"[Mesh:noexp] OR amphetamine[tiab] OR "Dextroamphetamine"[Mesh] OR dextroamphetamine[tiab] OR dexamphetamine[tiab] OR "d-amphetamine"[tiab] OR "mixed amphetamine salts"[tiab] OR "Lisdexamfetamine Dimesylate"[Mesh] OR lisdexamfetamine[tiab] OR atomoxetine[tiab] OR tomoxetine[tiab] OR "Clonidine"[Mesh] OR clonidine[tiab] OR guanfacine[tiab] OR viloxazine[tiab]) AND ("Spermatogenesis"[Mesh] OR "Spermatozoa"[Mesh] OR "Testis"[Mesh] OR "Epididymis"[Mesh] OR "Infertility, Male"[Mesh] OR sperm*[tiab] OR spermat*[tiab] OR testis[tiab] OR testes[tiab] OR testicular[tiab] OR epididym*[tiab] OR seminiferous[tiab] OR Leydig[tiab] OR Sertoli[tiab] OR "male fertility"[tiab] OR "male infertility"[tiab] OR "male reproductive toxicity"[tiab] OR "male reproductive function"[tiab] OR "male reproductive system"[tiab] OR ((male[tiab] OR males[tiab] OR paternal[tiab] OR sire[tiab] OR sires[tiab]) AND (fertil*[tiab] OR fecund*[tiab] OR mating[tiab] OR impregn*[tiab] OR "sexual maturation"[tiab] OR "preputial separation"[tiab]))) AND ((Animals[Mesh] NOT Humans[Mesh]) OR rat[tiab] OR rats[tiab] OR mouse[tiab] OR mice[tiab] OR murine[tiab] OR rabbit*[tiab] OR hamster*[tiab] OR "guinea pig"[tiab] OR "guinea pigs"[tiab] OR monkey*[tiab] OR primate*[tiab] OR canine[tiab] OR dog[tiab] OR dogs[tiab] OR pig[tiab] OR pigs[tiab] OR swine[tiab])

Embase

('methylphenidate'/exp OR methylphenidate:ti,ab OR dexmethylphenidate:ti,ab OR serdexmethylphenidate:ti,ab OR 'amphetamine'/exp OR amphetamine:ti,ab OR dextroamphetamine:ti,ab OR dexamphetamine:ti,ab OR 'd-amphetamine':ti,ab OR 'mixed amphetamine salts':ti,ab OR lisdexamfetamine:ti,ab OR atomoxetine:ti,ab OR tomoxetine:ti,ab OR 'clonidine'/exp OR clonidine:ti,ab OR guanfacine:ti,ab OR viloxazine:ti,ab) AND ('spermatogenesis'/exp OR 'spermatozoon'/exp OR 'testis'/exp OR 'epididymis'/exp OR 'male infertility'/exp OR sperm*:ti,ab OR spermat*:ti,ab OR testis:ti,ab OR testes:ti,ab OR testicular:ti,ab OR epididym*:ti,ab OR seminiferous:ti,ab OR leydig:ti,ab OR sertoli:ti,ab OR 'male fertility':ti,ab OR 'male infertility':ti,ab OR 'male reproductive toxicity':ti,ab OR 'male reproductive function':ti,ab OR 'male reproductive system':ti,ab OR ((male:ti,ab OR males:ti,ab OR paternal:ti,ab OR sire:ti,ab OR sires:ti,ab) AND (fertil*:ti,ab OR fecund*:ti,ab OR mating:ti,ab OR impregn*:ti,ab OR 'sexual maturation':ti,ab OR 'preputial separation':ti,ab))) AND ('animal experiment'/de OR rat:ti,ab OR rats:ti,ab OR mouse:ti,ab OR mice:ti,ab OR murine:ti,ab OR rabbit*:ti,ab OR hamster*:ti,ab OR 'guinea pig':ti,ab OR 'guinea pigs':ti,ab OR monkey*:ti,ab OR primate*:ti,ab OR canine:ti,ab OR dog:ti,ab OR dogs:ti,ab OR pig:ti,ab OR pigs:ti,ab OR swine:ti,ab)
