# Supplementary material for: Effects of ADHD Medications on Male Reproductive Function: A Systematic Review of In Vivo Mammalian Evidence
Source: Int J Mol Sci. 2026 Sep 23;27(19):8492. doi: 10.3390/ijms27198492 (PMC13665539; doi:10.3390/ijms27198492)
Supplement: Supplementary file 1 [file ijms-27-08492-s001.zip › Supplementary Material S8.pdf]

Supplementary Material 8. Ethics approval, funding, and conflict-of-interest reporting in the included studies

Study	Ethics approval	Funding or support	Conflicts of interest
**Abdollahifar et al., 2020**	Shahid Beheshti University of Medical Sciences Ethics Committee (IR.SBMU.MSP.REC.1398.31)	Vice Chancellor of Research, Shahid Beheshti University of Medical Sciences	No conflicts of interest declared
**Adriani et al., 2006**	NR	Joint NIH–ISS Project; Italian Ministry of Health; Dipartimento Nazionale Antidroga, Presidency of the Council of Ministers, Italy	NR
**Akhavan Rezayat et al., 2020**	Medical Ethics Committee, Mashhad University of Medical Sciences (ir.mums.fm.rec1395.315)	No funding/support reported by the authors	No conflicts of interest declared
**Basaki et al., 2021**	University of Tabriz (IR.TABRIZU.REC.1398.025)	Research Council, University of Tabriz	No conflicts of interest declared
**Beckman et al., 2008**	Good Laboratory Practice study; formal committee approval not reported	Industry-conducted study at Novartis Pharmaceuticals Corporation; no formal funding statement	NR; all authors were affiliated with Novartis Pharmaceuticals Corporation
**Cansu et al., 2011**	Animal Care Committee, Gazi University	Bayındır Hospital	NR
**Chaturapanich et al., 2002**	Mahidol University Research Project Reviewer Committee; CIOMS principles	Faculties of Science and Graduate Studies, Mahidol University	NR
**da Silva Júnior et al., 2014**	Federal University of São Paulo (UNIFESP 0016/2013)	CAPES, FAPESP, and CNPq (Brazil)	The authors reported nothing to disclose
**Fazelipour et al., 2012**	NR	NR	No conflicts of interest declared
**Fazelipour et al., 2014**	Institutional Animal Care and Use Committee; AAALAC-accredited facility	NR	NR
**Gomes et al., 2022**	CEUA/UNIFESP (5595139117)	FAPESP (2017/09712-4 and 2017/09725-9), CAPES/Brazil, and CNPq/PIBIC	No conflicts of interest declared
**Gomes et al., 2025**	CEUA/UNIFESP (2894060421)	CAPES/Brazil and CNPq/PIBIC; antibodies supported by FAPESP (2018/01473-3); APC funded by CAPES	No conflicts of interest declared
**Iqbal et al., 2019**	Institutional Ethics Review Committee	NR	No conflicts of interest declared
**Iqbal et al., 2025**	Ethical Review Committee, Baqai Medical University	No funding	No conflicts of interest declared
**Khoubbieh et al., 2023**	Yeditepe University Experimental Animal Research Ethics Committee (2021/01–12)	No funding	No relevant financial or non-financial interests declared
**Kianifard et al., 2013**	University of Tabriz; Research Ethical Committee standards of the Iranian Ministry of Health and Medical Education	NR	No competing interests declared
**Manjanatha et al., 2008**	NCTR Institutional Animal Care and Use Committee	FDA/NCTR–NICHD Interagency Agreement	NR
**Mattison et al., 2011**	NCTR Institutional Animal Care and Use Committee	NICHD/NIH–NCTR/FDA, NCTR–NIOSH, and ORISE–NCTR/FDA interagency agreements; NICHD intramural funds; NIH R01 HD013254	NR
**Montagnini et al., 2014**	UEL Ethics Committee for Animal Research (CEUA 16381.2012.45)	CAPES master’s fellowship	No conflicts of interest declared
**Raoofi et al., 2021**	Sabzevar University of Medical Sciences (IR.MEDSAB.REC.1399.080)	Cellular and Molecular Research Center, Sabzevar University of Medical Sciences (No. 98238)	No declarations of interest
**Rodrigues et al., 2025**	CEUA, Federal University of Viçosa (70/2018)	FAPEMIG PhD scholarship (BPD 00733–22)	No conflicts of interest declared
**Roustaee et al., 2024**	Shahid Beheshti University of Medical Sciences Animal Care and Use Committee (IR.SBMU.AEC.1401.082)	Men’s Health and Reproductive Health Research Center, Shahid Beheshti University of Medical Sciences	No competing interests declared

Note: Statements are reproduced or conservatively summarized from the source articles. NR indicates that no explicit statement was identified. For Beckman et al. (2008), the industry setting and author affiliations are reported separately because the article did not provide formal funding or conflict-of-interest declarations.

Abbreviations: AAALAC, Association for Assessment and Accreditation of Laboratory Animal Care International; APC, article processing charge; CAPES, Coordenação de Aperfeiçoamento de Pessoal de Nível Superior; CEUA, Ethics Committee on the Use of Animals; CIOMS, Council for International Organizations of Medical Sciences; CNPq, Conselho Nacional de Desenvolvimento Científico e Tecnológico; FAPEMIG, Fundação de Amparo à Pesquisa do Estado de Minas Gerais; FAPESP, São Paulo Research Foundation; FDA, US Food and Drug Administration; IACUC, Institutional Animal Care and Use Committee; NICHD, Eunice Kennedy Shriver National Institute of Child Health and Human Development; NIH, National Institutes of Health; NIOSH, National Institute for Occupational Safety and Health; NCTR, National Center for Toxicological Research; NR, not reported; ORISE, Oak Ridge Institute for Science and Education; UNIFESP, Federal University of São Paulo.
